# Electronegative Low-Density Lipoprotein Increases C-Reactive Protein Expression in Vascular Endothelial Cells through the LOX-1 Receptor

**DOI:** 10.1371/journal.pone.0070533

**Published:** 2013-08-08

**Authors:** Chih-Sheng Chu, Yu-Chen Wang, Long-Sheng Lu, Brian Walton, H. Ramazan Yilmaz, Roger Y. Huang, Tatsuya Sawamura, Richard A. F. Dixon, Wen-Ter Lai, Chu-Huang Chen, Jonathan Lu

**Affiliations:** 1 Division of Cardiology, Department of Internal Medicine, Kaohsiung Medical University Hospital, Kaohsiung Medical University, Kaohsiung, Taiwan; 2 Graduate Institute of Medicine, Kaohsiung Medical University, Kaohsiung, Taiwan; 3 Department of Medicine, Baylor College of Medicine, Houston, Texas, United States of America; 4 Department of Vascular & Medicinal Research, Texas Heart Institute, Houston, Texas, United States of America; 5 Division of Cardiology, Department of Internal Medicine, China Medical University Hospital, Taichung, Taiwan; 6 Department of Molecular Biology, Texas Heart Institute, Houston, Texas, United States of America; 7 Department of Medicine, National Research Institute of Chinese Medicine, Taipei, Taiwan; 8 Department of Vulnerable Plaque Research, Texas Heart Institute, Houston, Texas, United States of America; 9 Department of Basic Medical Sciences, Mevlana University, Konya, Turkey; 10 Department of Vascular Physiology, National Cerebral and Cardiovascular Center, Osaka, Japan; 11 Department of Molecular Cardiology, Texas Heart Institute, Houston, Texas, United States of America; 12 L5 Research Center, China Medical University Hospital, Taichung, Taiwan; 13 Graduate Institute of Medical Science, China Medical University, Taichung, Taiwan; Morehouse School of Medicine, United States of America

## Abstract

**Objectives:**

Increased plasma C-reactive protein (CRP) levels are associated with the occurrence and severity of acute coronary syndrome. We investigated whether CRP can be generated in vascular endothelial cells (ECs) after exposure to the most electronegative subfraction of low-density lipoprotein (LDL), L5, which is atherogenic to ECs. Because L5 and CRP are both ligands for the lectin-like oxidized LDL receptor-1 (LOX-1), we also examined the role of LOX-1.

**Methods and Results:**

Plasma LDL samples isolated from asymptomatic hypercholesterolemic (LDL cholesterol [LDL-C] levels, 154.6±20 mg/dL; n = 7) patients and normocholesterolemic (LDL-C levels, 86.1±21 mg/dL; *P*<0.001; n = 7) control individuals were chromatographically resolved into 5 subfractions, L1-L5. The L5 percentage (L5%) and the plasma L5 concentration ([L5]  =  L5% × LDL-C) in the patient and control groups were 8.1±2% vs. 2.3±1% (*P*<0.001) and 12.6±4 mg/dL vs. 1.9±1 mg/dL (*P*<0.001), respectively. In hypercholesterolemic patients treated with atorvastatin for 6 months (10 mg/day), [L5] decreased from 12.6±4 mg/dL to 4.5±1.1 mg/dL (*P* = 0.011; n = 5), whereas both [L5] and L5% returned to baseline levels in 2 noncompliant patients 3 months after discontinuation. In cultured human aortic ECs (HAECs), L5 upregulated CRP expression in a dose- and time-dependent manner up to 2.5-fold (*P*<0.01), whereas the least electronegative subfraction, L1, had no effect. DiI-labeled L1, internalized through the LDL receptor, became visible inside HAECs within 30 seconds. In contrast, DiI-labeled L5, internalized through LOX-1, became apparent after 5 minutes. L5-induced CRP expression manifested at 30 minutes and was attenuated by neutralizing LOX-1. After 30 minutes, L5 but not L1 induced reactive oxygen species (ROS) production. Both L5-induced ROS and CRP production were attenuated by ROS inhibitor N-acetyl cysteine.

**Conclusions:**

Our results suggest that CRP, L5, and LOX-1 form a cyclic mechanism in atherogenesis and that reducing plasma L5 levels with atorvastatin disrupts the vascular toxicity of L5.

## Introduction

Atherosclerosis is an inflammatory disease caused by the deposit of lipids within the vessel wall, which can be induced by multiple factors including age, smoking, endothelial dysfunction, and reactive oxygen species (ROS) [1]. Among numerous inflammatory markers, C-reactive protein (CRP), predominantly produced by hepatocytes [2], [3] under the stimulation of inflammatory cytokines such as interleukin 6 (IL-6), is one of the most well-validated markers and serves as an independent predictor in various stages of atherosclerosis [4]. Clinically, elevated CRP levels predict higher cardiovascular event rates [5]. In patients with acute coronary syndrome, an elevated CRP level has also been shown to be a good predictor of morbidity and mortality [4]. In addition to being an acute-phase inflammatory marker, CRP is also considered the causative factor of low-grade vascular inflammation in atherosclerosis [6], [7]. Reports suggest that plasma CRP levels may be useful for guiding lipid-lowering therapy in individuals who appear to be at low risk for cardiovascular disease according to the traditional Framingham Risk Score [8]. C-reactive protein can detrimentally interact with vascular endothelium and contribute to atherothrombosis by inducing monocyte–endothelium adhesion, promoting atherogenic low-density lipoprotein (LDL) cholesterol accumulation in macrophages, increasing ROS production, decreasing endothelial nitric oxide production, triggering platelet aggregation, and making atherosclerotic plaque prone to rupture [9]. Venugopal and colleagues reported that cytokine stimulation of endothelial cells could result in a much higher local CRP concentration than that observed in plasma and that this higher concentration may contribute to inflammatory and atherogenic effects [10].

In addition to CRP, LDL has been confirmed in several clinical trials to have a role in cardiovascular events [11]–[13], but the specific LDL implicated in atherogenesis remains unknown. Plasma LDL can be chromatographically divided into 5 subfractions on the basis of its surface electric charge [14], [15]. The most electronegative subfraction, L5, is the only one capable of inducing monocyte–endothelial cell adhesion, endothelial cell apoptosis, impaired endothelial progenitor cell differentiation, and accelerated endothelial progenitor cell senescence [14], [16]–[18]. Plasma L5 levels are elevated in patients with high cardiovascular risks such as hypercholesterolemia, type 2 diabetes mellitus, and smoking [14], [16], [19]. In addition, we have recently shown that plasma L5 levels are drastically elevated in patients with ST-elevation myocardial infarction [20]. Researchers recently identified CRP in the supernatant of human aortic endothelial cells (HAECs) treated with oxidized LDL [21]. Interestingly, L5 and CRP are both ligands for the lectin-like oxidized LDL receptor-1 (LOX-1) [18], [22], a class E scavenger receptor that was first cloned from bovine aortic endothelial cells [23]. LOX-1 is the major endothelial cell receptor for oxidized LDL in humans and in various animal models, and LOX-1 is believed to play a role in the pathogenesis of atherosclerosis [24]. In human atherosclerotic lesions, LOX-1 is expressed in intimal smooth muscle cells, lipid-laden macrophages, and plaque neovasculature [25]. A recent study showed that statins, which are HMG-CoA (3-hydroxy-3-methyl-glutaryl-CoA) reductase inhibitors, inhibited LOX-1 expression in atheromas of Watanabe heritable hyperlipidemic rabbits [26], suggesting a potential relationship between statins, L5, and CRP. However, it is unknown whether L5 has effects similar to those of oxidized LDL, and a correlation between the roles of CRP and L5 in atherosclerosis has not been established.

Statins have been found to be beneficial for slowing atherosclerosis progression throughout the cardiovascular disease continuum by means of a mechanism beyond their lipid-lowering effects. In patients with acute coronary syndrome, statin drug therapy lowered both plasma LDL and CRP levels and provided early clinical benefits [27], [28]. In the ASCOT-LLA (Anglo-Scandinavian Cardiac Outcomes Trial–Lipid-Lowering Arm) study, atorvastatin therapy reduced major cardiovascular events in hypertensive patients with or without dyslipidemia [29]. The pleiotropic effects of statin drugs were believed to be associated with their anti-inflammatory role. However, the effect of statin drugs on the presence of electronegative LDL has not been reported. We hypothesized that L5 upregulates the production of CRP in HAECs and that statins lower plasma L5 levels, thereby reducing the cytotoxic effects of L5.

## Results

### Effects of Atorvastatin on Plasma L5 Levels

**Figure 1:**
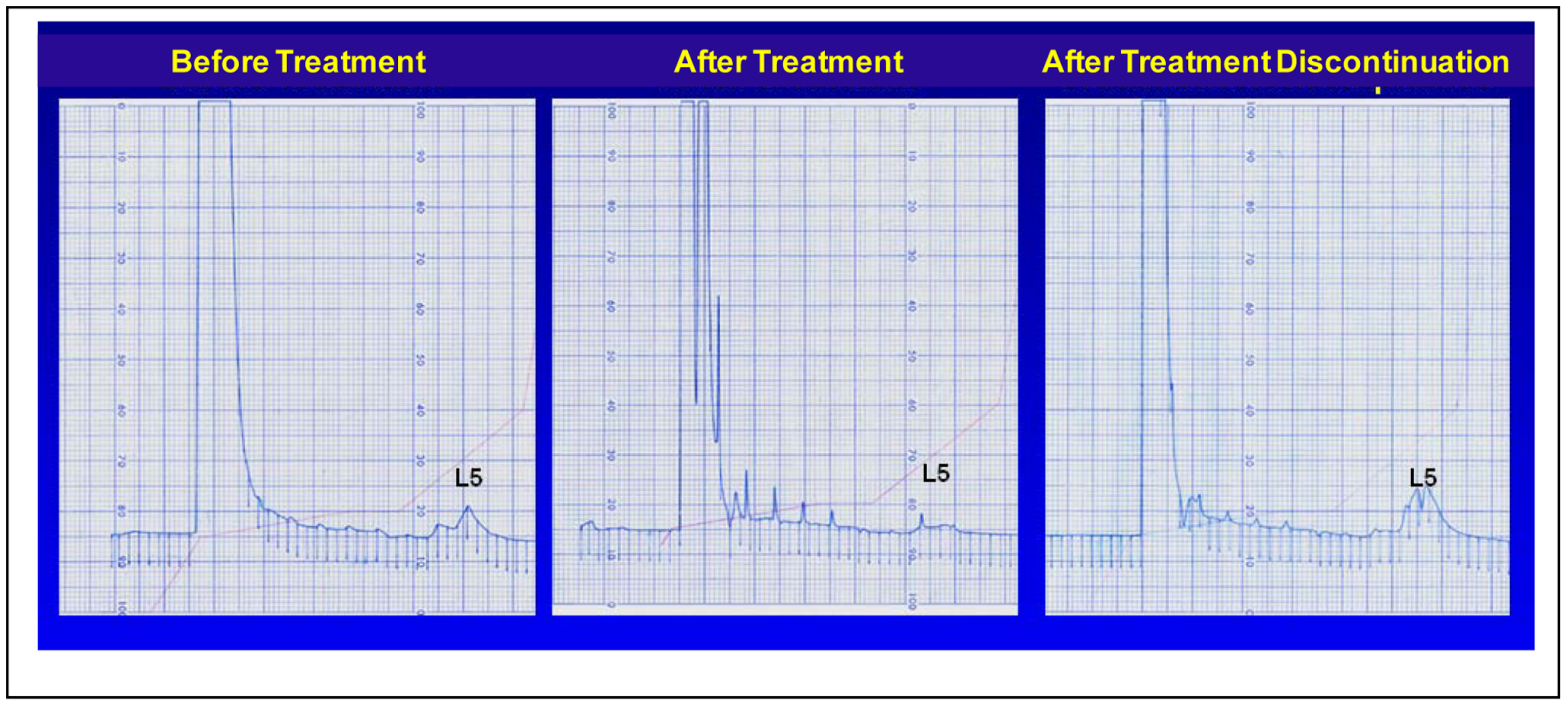
Anion-exchange chromatography of low-density lipoprotein (LDL) from a hypercholesterolemic patient. Chromatograms showing the L5 percentage before treatment (left), after 3 months of atorvastatin treatment (10 mg/day; center), and 3 months after the discontinuation of treatment (right) in hypercholesterolemic patient (baseline plasma LDL-cholesterol level, 226 mg/dL). Treatment with atorvastatin for 3 months reduced the level of L5, and discontinuation of treatment for 3 months resulted in the recurrence of elevated L5.

The baseline characteristics of healthy control subjects (n = 7) and hypercholesterolemic patients (n = 7) are shown in Table 1. The mean L5 percentage at baseline was significantly higher in hypercholesterolemic patients than in control subjects (8.1±2% vs. 2.3±1%; *P*<0.001; Table 1). The baseline characteristics of hypercholesterolemic patients before and after atorvastatin treatment (n = 5) are shown in Table 2. After 6 months of statin treatment, the L5 percentage in hypercholesterolemic patients was significantly reduced to 4.5±0.8% (*P* = 0.022; Table 2), and the total LDL cholesterol (LDL-C) concentration was reduced from 154.6±20 mg/dL at baseline to 100±12 mg/dL (Table 2). In 2 patients who discontinued statin use after 3 months of treatment, the L5 percentage returned to pretreatment levels 3 months after discontinuation; the results of fast protein liquid chromatography analysis of L5 in 1 patient before treatment, after 3 months of treatment, and 3 months after the discontinuation of treatment are shown in Figure 1. The data for the other patient who discontinued statin therapy were similar (data not shown). The L5 concentration ([L5]) was also calculated as an indicator of potential atherogenic capacity in the plasma. By definition, [L5] is derived from the multiplication of the L5 percentage and the LDL-C concentration (in mg/dL). At baseline, the [L5] was higher in hypercholesterolemic patients than in control subjects (12.6±4 mg/dL vs. 1.9±1 mg/dL) and was reduced to 4.5±1.1 mg/dL after atorvastatin treatment. Thus, atorvastatin quantitatively reduced both the total plasma LDL-C level and the [L5], and discontinuation of statin therapy resulted in the recurrence of elevated L5 levels.

**Table 1:** Characteristics of Patients with Hypercholesterolemia and Healthy Control Subjects Before Atorvastatin Treatment.

Variable^a^	Control Subjects (n = 7)	Patients (n = 7)	P-value
Age (y)	38±7	50±7	0.006
Sex (F/M)	2/5	2/5	1
BMI (kg/m^2^)	23±4	40±6	0.001
Cholesterol (mg/dL)	155.3±30	223.9±39	0.003
TG (mg/dL)	59.0±23	158.3±50	0.001
HDL-C (mg/dL)	57.6±12	47.0±13	0.15
VLDL-C (mg/dL)	11.8±5	31.6±10	0.001
LDL-C (mg/dL)	86.1±21	154.6±20	<0.001
L5 (%)	2.3±1	8.1±2	<0.001
[L5]^b^ (mg/dL)	1.9±1	12.6±4	<0.001

a Data are shown as the number or as the mean ± standard deviation. BMI, body mass index; TG, triglyceride; HDL-C, high-density lipoprotein cholesterol; VLDL-C, very-low-density lipoprotein cholesterol; LDL-C, low-density lipoprotein cholesterol.

b [L5]  =  L5% × LDL-C.

**Table 2:** Characteristics of Patients with Hypercholesterolemia Before and After Atorvastatin Treatment.

Variable^a^	Patients Before Statin^c^ (n = 5)	Patients After Statin^c^ (n = 5)	P-value
Age (y)	50±7	50±9	-
Sex (F/M)	1/4	1/4	-
BMI (kg/m^2^)	40±6	43±5	-
Cholesterol (mg/dL)	223.9±39	177.4±21	0.04
TG (mg/dL)	158.3±50	131.8±30	0.71
HDL-C (mg/dL)	47.0±13	50.6±9	0.52
VLDL-C (mg/dL)	31.6±10	26.6±6	0.75
LDL-C (mg/dL)	154.6±20	100.0±12	0.002
L5 (%)	8.1±2	4.5±0.8	0.02
[L5]^b^ (mg/dL)	12.6±4	4.5±1.1	0.01

a Data are shown as the number or as the mean ± standard deviation. BMI, body mass index; TG, triglyceride; HDL-C, high-density lipoprotein cholesterol; VLDL-C, very-low-density lipoprotein cholesterol; LDL-C, low-density lipoprotein cholesterol.

b [L5]  =  L5% × LDL-C.

c Two patients discontinued statin therapy after 3 months of treatment and were not included in this analysis.

### Western Blot and Enzyme-Linked Immunosorbent Assay Analysis of Endothelial CRP Expression

**Figure 2:**
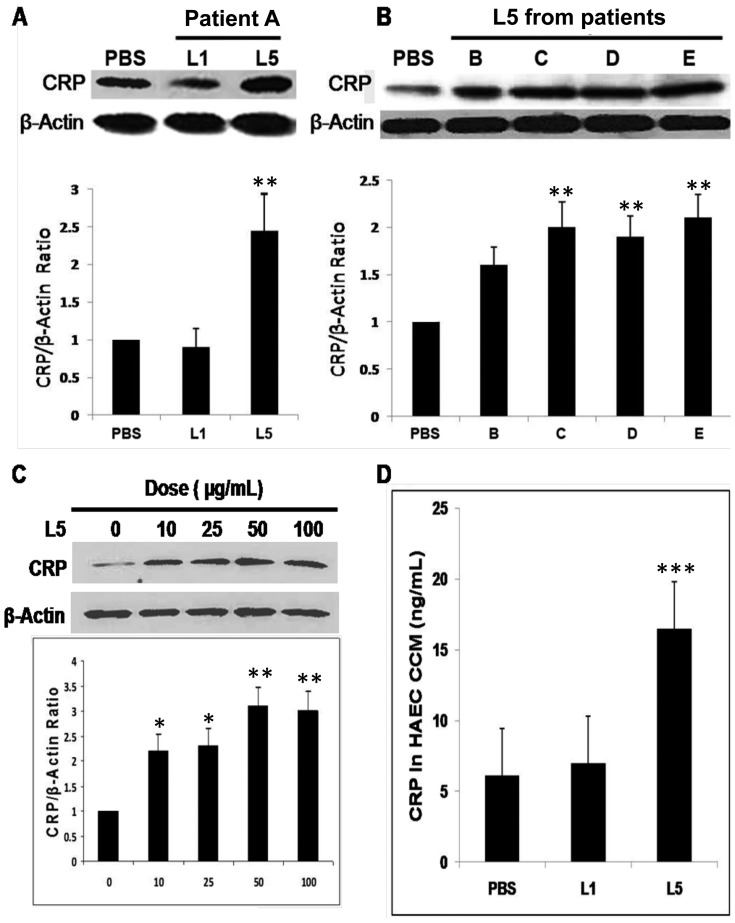
Analysis of C-reactive protein (CRP) expression by using Western blot and enzyme-linked immunosorbent assay (ELISA). (A, B) C-reactive protein expression in human aortic endothelial cells (HAECs) treated with L5 from 5 hypercholesterolemic patients (patients labeled as A–E). L5 increased endothelial CRP expression by up to 2.5-fold more than did L1. (C) L5 treatment (10–100 µg/mL) increased endothelial CRP expression in a dose-dependent manner. (D) ELISA analysis of CRP in the conditioned culture medium (CCM) of HAECs treated with phosphate-buffered saline (PBS), L1, or L5. For all experiments, n = 3, and bars shown in graphs represent standard deviation. *P<0.05, **P<0.01, ***P<0.001 vs. PBS or untreated control.

As previously reported, the amount of L5 from healthy control subjects was too low for further analysis [14]. We verified the cytotoxicity of L5 isolated from hypercholesterolemic patients by treating HAECs with 50 μg/mL of each LDL subfraction (L1-L5) for 24 hours; only L5-treated HAECs showed notable evidence of apoptosis (Fig. S1). To examine whether L5 affects CRP expression, HAECs were treated with phosphate-buffered saline (PBS), L1, or L5 (50 μg/mL each) and lysed, followed by analysis of CRP expression by using a Western blot assay. L5 isolated from hypercholesterolemic patients upregulated endothelial CRP expression up to 2.5-fold higher than did PBS, whereas L1 had no effect on CRP expression (Fig. 2A and 2B). Furthermore, L5 increased endothelial CRP expression in a dose-dependent manner. When HAECs were treated with L5 concentrations of 10 to 100 μg/mL for 24 hours, CRP levels peaked in cells treated with 50 μg/mL L5; however, 100 μg/mL L5 did not induce any further increase in CRP levels (Fig. 2C). Moreover, enzyme-linked immunosorbent assay (ELISA) results showed that CRP secretion was 2- to 3-fold higher in the conditioned culture medium (CCM) of L5-treated HAECs than in the CCM of L1- or PBS-treated HAECs (Fig. 2D).

### Total ROS Production in HAECs and Oxidization Analysis of L5

**Figure 3:**
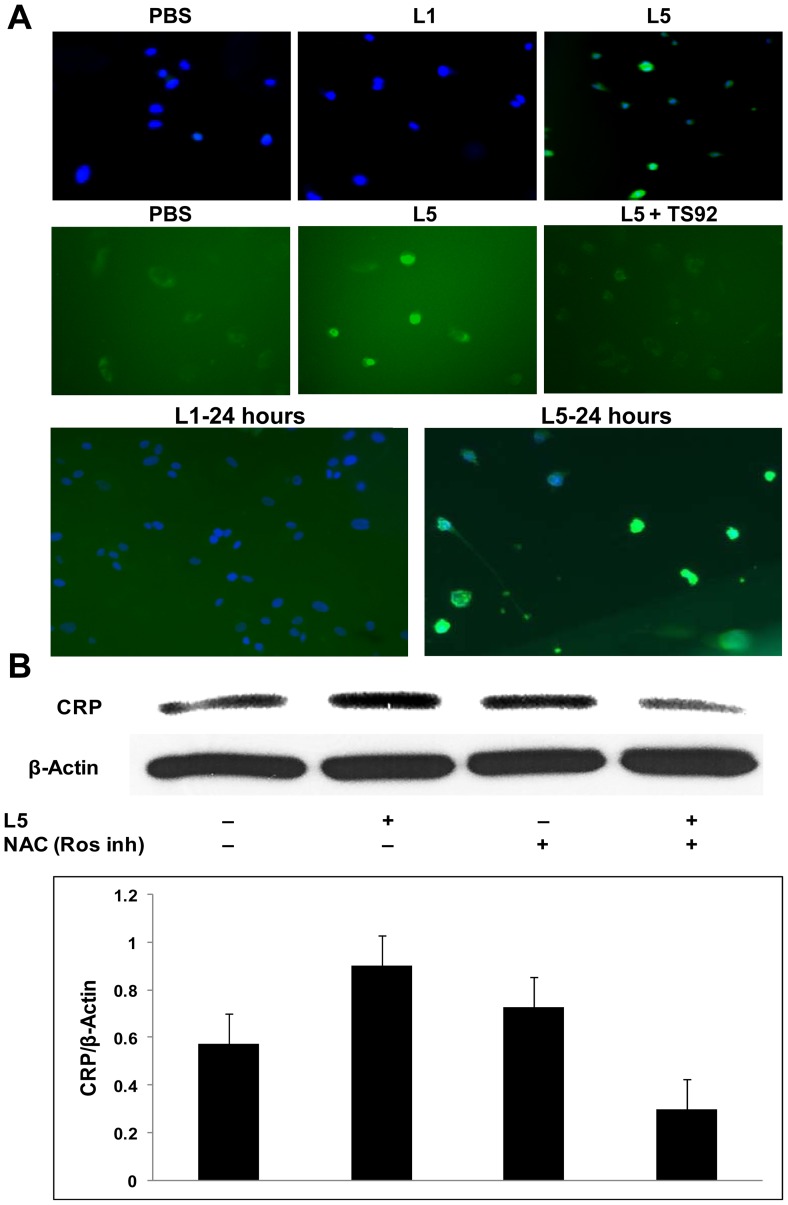
Detection of total reactive oxygen species (ROS) in human aortic endothelial cells (HAECs). (A) L5 (50 µg/mL) from patients increased ROS production (green) in HAECs after 20 minutes (top and middle row) and after 24 hours (bottom row). Compared to the phosphate-buffered saline (PBS) control, L1 treatment had no effect. Staining with 4′,6-diamidino-2-phenylindole (DAPI) (blue) indicated HAEC apoptosis 24 hours after L5 (50 µg/mL) exposure. The pretreatment of cells with TS92 attenuated the L5-induced ROS increase after 20 minutes (middle row). (B) Blocking the production of ROS by adding ROS inhibitor N-acetyl cysteine (NAC) attenuated the L5-induced increase in CRP levels. For all experiments, n = 3, and bars shown in graphs represent standard deviation. *P<0.05, **P<0.01 vs. untreated control.

We examined the production of ROS by using fluorescence microscopy and found that HAECs treated for 20 minutes with L5 (50 μg/mL) isolated from hypercholesterolemic patients stimulated the production of ROS, whereas L1 (50 μg/mL) had no effect on ROS production (Fig. 3A). To determine whether L5 continued to stimulate ROS production over time, HAECs were also incubated with L5 for 24 hours, the same incubation period used for many of the Western blots. After 24 hours, ROS production in HAECs remained elevated (Fig. 3A). To further investigate the pathway of L5-induced ROS production, we neutralized the L5 receptor, LOX-1, by using the TS92 antibody. Western blot analysis showed that L5-induced production of ROS was diminished in HAECs pretreated with 10 μg/mL TS92 (Fig. 3A). Furthermore, in HAECs pretreated with the ROS inhibitor N-acetyl cysteine (5 mM), L5-induced CRP expression was diminished (Fig. 3B). Another oxidative stress marker, superoxide dismutase 1 (SOD1), was also augmented by L5 after 24 hours treatment (Fig. S2A). Interestingly, thiobarbituric acid reactive substances (TBARs) assays revealed that, compared with oxLDL, L1 and L5 samples from 4 patients all had very low malondialdehyde (MDA) values, similar to those for native LDL [30] (Fig. S2B).

### Analysis of the L5-induced CRP Pathway

**Figure 4:**
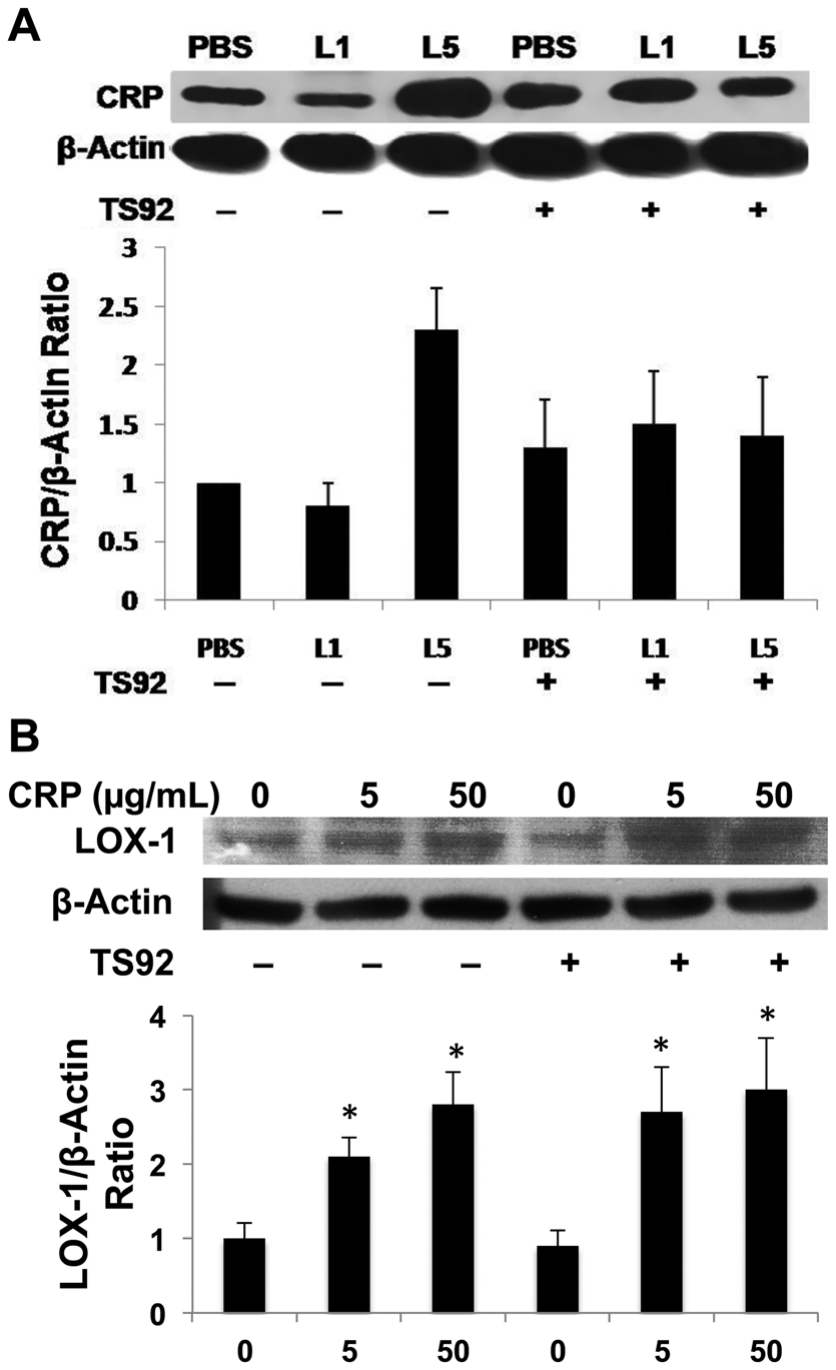
Effects of lectin-like oxidized LDL receptor-1 (LOX-1) inhibition in human aortic endothelial cells (HAECs). (A) HAECs were pretreated with LOX-1 neutralizing antibody TS92 before the introduction of L1 or L5 into cell culture. (B) HAECs were pretreated with TS92 before the introduction of recombinant human CRP (5 or 50 µg/mL) into cell culture. For all experiments, n = 3, and bars shown in graphs represent standard deviation. *P<0.05 vs. untreated control.

LOX-1 is not only the receptor for L5, but it also serves as a receptor for CRP [18], [31]. By manipulating the L5/LOX-1 interaction, we investigated the pathway through which L5 induces an increase in CRP levels. Pretreatment of HAECs with TS92 (10 μg/mL) to block the LOX-1 receptor significantly attenuated the L5-induced augmentation of CRP levels (Fig. 4A). Furthermore, by adding increasing concentrations of commercially synthesized recombinant human CRP to HAECs, LOX-1 expression was augmented in a dose-dependent manner. However, blocking LOX-1 with TS92 did not attenuate the recombinant CRP-induced augmentation of LOX-1 expression (Fig. 4B). Moreover, in HAECs treated with 50 μg/mL L5, we observed an increase in the inflammatory markers TNFα (as previously described [18]), p-p38, and NF-κB (Fig. S3).

### Internalization of L1 and L5 by HAECs

**Figure 5:**
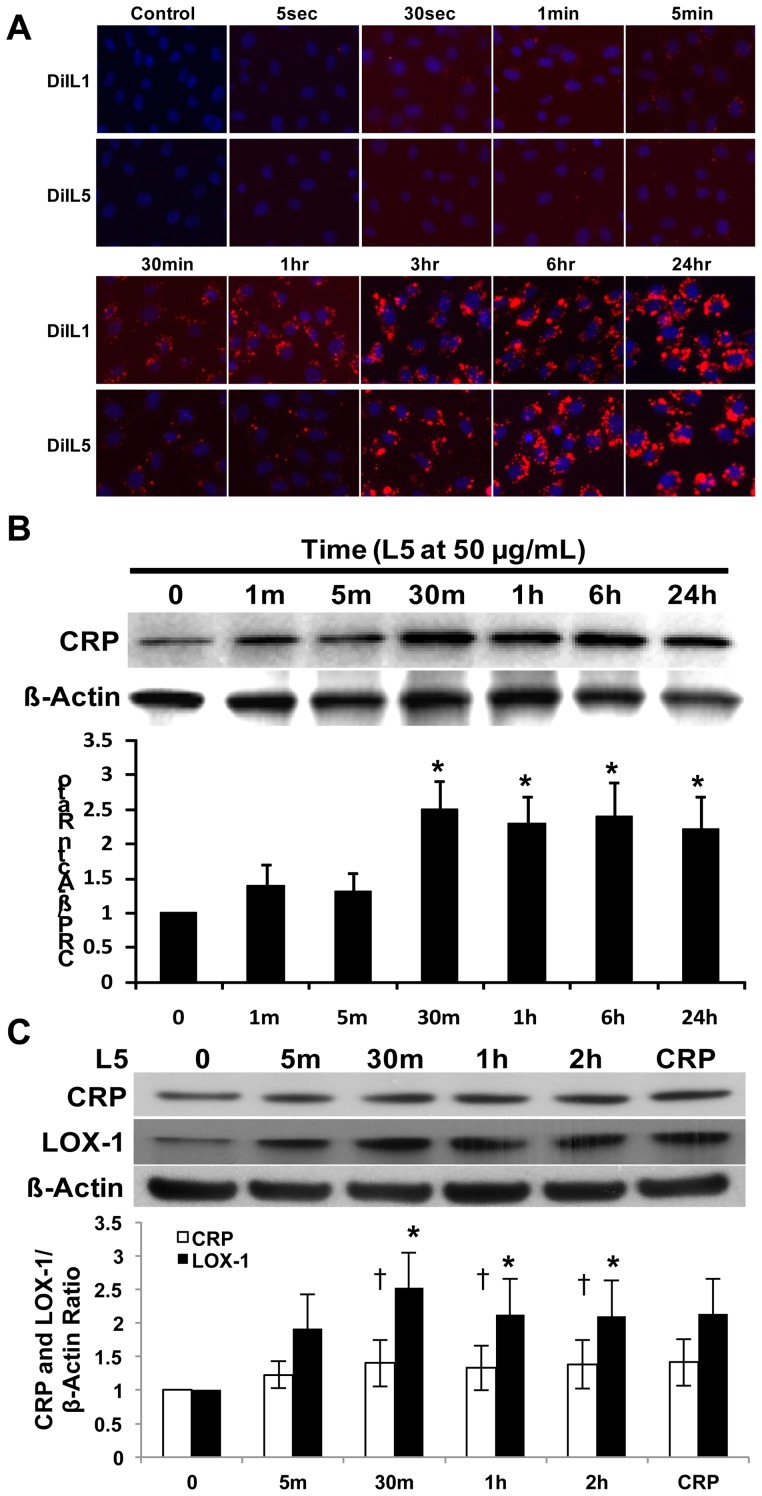
Time-course analysis of L1 and L5 internalization by human aortic endothelial cells (HAECs) and L5-induced C-reactive protein (CRP) expression. (A) Results of fluorescence microscopy showing that DiI-L1 and DiI-L5 (each red) were internalized by HAECs at different time points. The nuclei of HAECs were co-stained with 4′,6-diamidino-2-phenylindole (DAPI) (blue). (B) Western blot showing that L5 augmented endothelial CRP expression in a time-dependent manner. Time point 0 represents the phosphate-buffered saline (PBS) control. *P<0.05 vs. PBS-treated control. (C) L5 deprivation study showed that internalized L5 continued to induce CRP and lectin-like oxidized receptor-1 (LOX-1) expression 24 hours after replacing L5 conditioned culture media with fresh EGM2 media. L5 exposure times are shown. HAECs were incubated with recombinant CRP for 2 hours as a positive control. Time point 0 represents the PBS control. For all experiments, n = 3, and bars in graphs represent standard deviation. †*P*<0.05 vs. PBS-treated CRP control, *P<0.05 vs. PBS-treated LOX-1 control.

To analyze the internalization of L1 and L5 and the L5-CRP pathway, we added L1 and L5 labeled with fluorescent lipophilic dye DiI (1,1′-dioctadecyl-3,3,3′,3′-tetramethylindocarbocyanine perchlorate) to passage 4 HAECs. To characterize the time course for L1 and L5 internalization, we examined the HAECs by using fluorescence microscopy at 10 time points during a 24-hour period. The HAECs started to internalize L1 as early as at 30 seconds, whereas L5 was detected after 5 minutes. At 30 minutes, the internalized level of L1 was markedly higher than that of L5, confirming our previous finding that L1 and L5 are internalized by endothelial cells at a different pace through separate receptors [18] and, thus, different pathways (Fig. 5A). When we examined CRP expression, a time-dependent study revealed that L5 (50 μg/mL) augmented CRP expression as early as at 1 minute and peaked at 30 minutes (Fig. 5B). To rule out the possibility of secreted CRP's influencing LOX-1, the CCM of HAECs containing L5 was removed and replaced with fresh EGM2 medium (Lonza, Allendale, NJ, USA) after each time point. After the HAECs were continually cultured in fresh EGM2 for an additional 24 hours, Western blot analysis showed that CRP expression remained increased in HAECs after 24 hours (Fig. 5C). These results may indicate that although the internalization of L5 starts at 5 minutes, the amount internalized reaches significance at around 30 minutes and is critical for triggering a perceptible elevation in CRP levels. However, LOX-1 levels were elevated earlier, at 5 minutes, and increased in a time-dependent manner, peaking at 30 minutes (Fig. 5C). These results provide evidence that L5-LOX-1 signaling occurs upstream of L5-CRP and that LOX-1 may play a critical role in the L5-CRP pathway.

## Materials and Methods

### Study Design

All procedures were approved by Baylor College of Medicine's institutional review board. All participants provided written consent, and ethics committees approved the consent procedure. Individuals included in this observational study were either healthy control subjects or hypercholesterolemic patients, according to the coronary heart disease risk classification guidelines established by the Adult Treatment Panel III (ATP III) of the National Cholesterol Education Program (NCEP) [11], [32]. All patients and control subjects were admitted through the clinic of the Behavioral Medicine Research Center at Baylor College of Medicine in Houston, Texas. We recruited 28 subjects between 35 and 64 years of age, an age range similar to the criteria used in the ARIC (Atherosclerosis Risk in Communities) study (45–64 years of age) [33]. Plasma high-sensitivity CRP, high-density lipoprotein cholesterol, total cholesterol, triglyceride, LDL-C, very-low-density lipoprotein cholesterol, and glucose levels of the individuals studied were measured at Baylor Clinic, Baylor College of Medicine, during the initial screening and at each time point thereafter. Patients who had at least one of the following conditions were excluded from the study: 1) a positive history of stroke or recent coronary artery disease events (within 6 months); 2) concomitant use of lipid-lowering drugs including statins, ezetimibe, fibrates, niacin, and bile acid-binding resins; 3) current use of antihypertensive drugs; 4) use of a special diet for weight loss or lipid modification, such as a diet containing a red yeast rice supplement; 5) use of fish oil; 6) smoking; or 7) pregnant or planning to conceive.

Seven adult patients with asymptomatic hypercholesterolemia (M:F = 2:5; age, 50±7 years; body mass index, 40±6) met our criteria and were included in this study. Seven healthy control subjects (M:F = 2∶5; age, 38±7 years; body mass index, 23±4) were selected from among volunteers through the same center. The ethnic distribution of all study individuals followed the population composition in the Greater Houston Area. Blood samples were obtained from the study individuals for the isolation of LDL, described below.

### Atorvastatin Treatment

As part of a standard treatment regimen independent of this study, all hypercholesterolemic patients were treated with atorvastatin (10 mg/day) for 6 months. At the end of the 6-month treatment, blood samples were collected from all patients for follow-up; the same blood parameters stated above were determined for comparison to baseline levels. Two patients became noncompliant with this treatment after 3 months; their blood samples were collected and analyzed after 3 months of treatment and again 3 months after treatment discontinuation.

### Subfractionation of LDL

After plasma samples were collected from the study subjects, each individual's LDL was isolated according to its density gradient (1.019 to 1.063 g/mL) [34], [35]. Special precaution was taken to avoid coagulation and ex vivo oxidation [36]. To prevent contamination and experimental oxidation, 50 mU/mL of aprotinin, 1% ampicillin/streptamycin, and 0.5 mmol/L ethylenediaminetetraacetic acid (EDTA) were added to the human plasma samples immediately after collection. L1 to L5 subfractions were resolved with fast protein liquid chromatography by using an anion-exchange column (BioRad, Hercules, CA, USA) according to the method previously described [15], [34]. To confirm the cytotoxicity of L5, HAECs were treated with 50 ug/mL each of L1-L5 or phosphate-buffered saline for 24 hours, and apoptosis was examined by using Hoechst 33342 nuclear staining. The L5 percentage was calculated by using Unicorn 5.20 software designed by GE. For each patient, the plasma L5 concentration was calculated by multiplying the L5 percentage with the LDL-C concentration. The final protein concentrations of L1-L5 in solution were determined by using the Lowry method. TBARS assays were performed to measure oxidative lipid modification, as previously described [30].

### Cell Culture

Primary HAECs (passage number <6) (Lonza) were cultured in EGM2 medium (Lonza) for cell studies [37]. The HAECs were cultured in 12-well plates until the cells reached 60%–80% confluency before being used in any cellular assay.

### Western Blot Analysis

For all Western blot analyses, HAECs were lysed in NETN (150 mM NaCl, 20 mM Tris-HCl [pH 8.0], 0.5 mM EDTA, 0.5% Nonidet P-40) buffer, and protein extract concentrations were measured by using a BCA assay (Pierce, Rockford, IL, USA). For each sample lysate, 10 µg was used for sodium dodecyl sulfate polyacrylamide gel electrophoresis (SDS-PAGE) (4%–20% gels; Invitrogen, San Diego, CA, USA) [35]. The separated proteins were transferred to nitrocellulose paper (BioRad) and blocked with SuperBlock (Pierce, Rockford, IL, USA). Primary antibodies used for Western blots included anti–SOD-1 (#89786; Abcam, Cambridge, MA, USA), anti-TNFα (#MAB210; R&D Systems, Minneapolis, MN, USA), anti-p-38 (#9211; Cell Signaling, Boston, MA, USA), anti–NF-κB (#91626; Abcam, Cambridge, MA, USA), anti–LOX-1 (R&D Systems, Minneapolis, MN, USA), anti-CRP (#50861; Abcam, Cambridge, MA, USA), and monoclonal mouse anti-β-actin (Sigma-Aldrich, St. Louis, MO, USA), which was used for normalization. Secondary antibodies included anti-rabbit, -goat, and -mouse IgG antibodies conjugated to horseradish peroxidase (Amersham Biosciences, Sunnyvale, CA, USA). Signals were recorded on Kodak film by using chemiluminescent reagents (Enhanced Chemiluminescence Plus; Amersham Biosciences). The results were normalized to those of β-actin by means of ImageJ quantification (n = 3).

### Analysis of CRP and LOX-1 Expression

First, we determined the effects of each patient's L1 and L5 on CRP expression. In this experiment, 50 μg/mL L1, 50 μg/mL L5 (from each of 5 patients), or PBS (control) was added to the appropriate wells. The HAECs were then incubated for 24 hours at 37°C, and SDS-PAGE and Western blotting were performed as outlined above. To examine the role of the LOX-1 receptor in CRP expression, we pretreated HAECs with LOX-1 neutralizing antibody TS92 (10 μg/mL) for 1 hour before L1 and L5 were added. An L5 dose-response analysis was performed by exposing HAECs to PBS only or L5 at 10, 25, 50, and 100 μg/mL for 24 hours at 37°C, followed by the detection of CRP expression by SDS-PAGE and Western blotting. To measure secreted CRP levels in endothelial cell medium, we used the human CRP ELISA kit (Invitrogen). After the HAECs were treated with L1, L5, or PBS, the CCM from each sample was collected and filtered. The human CRP level in each CCM sample was then measured according to the protocol provided by the manufacturer. In addition, an L5 time-course study was performed by exposing HAECs to PBS (indicated as time point 0) or L5 (50 μg/mL) for 1 minute, 5 minutes, 30 minutes, 1 hour, 6 hours, and 24 hours at 37°C, followed by the detection of CRP expression by SDS-PAGE and Western blotting.

To determine the effects of CRP on LOX-1 expression, HAECs were incubated with PBS buffer or 5 or 50 µg/mL of recombinant human CRP for 24 hours at 37°C, followed by SDS-PAGE and Western blotting. Recombinant human CRP was purchased from Sigma-Aldrich (# C1617). To determine the role of the LOX-1 receptor, this experiment was then repeated as above with the exception that TS92 (10 μg/mL) was added to the cells for 1 hour before the addition of CRP.

To determine whether the effects of L5 on CRP and LOX-1 expression continued after L5 removal, HAECs were incubated with PBS (indicated as time point 0) or L5 (50 µg/mL) at 37°C for 5 minutes, 30 minutes, 1 hour, or 2 hours. HAECs were incubated with 10 µg/mL recombinant CRP for 2 hours as a positive control. After each exposure period, the cells were washed with PBS and incubated for another 24 hours at 37°C in L5-free EGM2 medium. SDS-PAGE and Western blotting were then performed as outlined above.

### Detection of ROS

To assess the effects of L1 and L5 on ROS production, HAECs were incubated with 50 µg/mL L1, 50 µg/mL L5, or PBS for either 20 minutes or 24 hours at 37°C. A Total ROS Detection Assay Kit (#ENZ051010; Enzo Life Sciences, Farmingdale, NY, USA) was used to detect endogenous ROS production in endothelial cells according to the manufacturer's instructions. Cells were observed by using fluorescence microscopy (Zeiss Axiovert 200 Inverted Fluorescence Microscope; Carl Zeiss Microscopy GmbH, Jena, Germany). Cells were costained with 4′,6-diamidino-2-phenylindole (DAPI). In a separate experiment designed to assess the role of LOX-1 in L5-induced ROS production, TS92 (10 µg/mL) was added to the HAECs 1 hour before L5 treatment to neutralize LOX-1. To study the role of ROS production in L5-induced CRP expression, we pretreated HAECs with 5 mM NAC (provided in the Total ROS Detection Assay Kit) 60 minutes before L5 was added.

### Time-Course Study of L1 and L5 Internalization by HAECs

Purified L1 and L5 were labeled with fluorescent lipophilic dye 1,1′-dioctadecyl-3,3,3′,3′-tetramethylindocarbocyanine perchlorate (DiI-C18, Invitrogen). In brief, DiI-labeled L1 and L5 were freshly prepared before use, as previously described [38]. L1 and L5 were diluted to 1 mg/mL with PBS and incubated with 80 µmol/L DiI at 37°C overnight. DiI-L1 and DiI-L5 were then purified by using ultracentrifugation at a density of 1.063 g/mL and were subsequently dialyzed against PBS-EDTA (0.5 mmol/L), being protected from light at all steps. DiI-L1 and DiI-L5 were purified by using a PD-10 desalting column (GE Healthcare, Piscataway, NJ, USA) and were added into the HAECs. DiI-labeled L1 and L5 were normalized by protein content and added to the primary HAECs at a concentration of 50 µg/mL. PBS was used as a control. The HAECs were then incubated at 37°C for the following time intervals: 5 seconds, 30 seconds, 1 minute, 5 minutes, 30 minutes, 1 hour, 3 hours, 6 hours, and 24 hours. After treatment, the cells were washed with PBS. Cellular internalization was observed by using a Zeiss Axiovert Inverted 200 fluorescence microscope to record the positions of DiI-L1 and DiI-L5 with respect to bright field images in overlay.

### Statistical Analysis

All experiments were repeated 3 times. Continuous variables were expressed as the mean ± standard deviation for normally distributed variables. Differences between the mean values for the control subjects versus the patients before statin therapy were analyzed by using a Student *t* test. A paired *t* test was used to determine the statistical difference between the values of the serial examination before and after statin therapy. A 2-tailed *P* value of <0.05 was considered significant. All analyses were performed by using SPSS 12.0 software (SPSS Inc., Chicago, IL, USA).

## Discussion

Electronegative LDL is known for its pro-atherogenic properties and strong correlation to the risk of cardiovascular events. In the present study, we found that the L5 percentage of total LDL levels was increased in patients with asymptomatic hypercholesterolemia and decreased after 6 months of atorvastatin treatment. Furthermore, in 2 patients who discontinued atorvastatin therapy after 3 months, the L5 percentage returned to pretreatment levels 3 months later. We also observed that L5 induced the production of ROS within 20 minutes of exposure and directly increased endogenous CRP levels via LOX-1 in HAECs within 30 minutes. Our results suggest that CRP, L5, and LOX-1 form a cyclic mechanism in atherogenesis and that reducing plasma L5 with atorvastatin disrupts the vascular toxicity of L5.

### L5/LOX-1/CRP Cyclic Mechanism

Elevation of CRP levels is normally a result of acute-phase inflammation [39]. Our findings suggest that L5 may initiate this pro-inflammatory process and worsen atherosclerosis. We previously showed that L5 promotes the expression of endothelial tumor necrosis factor alpha (TNF-α) – a positive acute-phase protein that contributes to the response to inflammatory stimulation – and that L5 enhances LOX-1 receptor expression in HAECs [18]. In the present study, we found that L5 increased intracellular CRP expression in HAECs, in a dose- and time-dependent manner, as early as 30 minutes after L5 exposure. Moreover, L5 dramatically increased the secretion of CRP in HAECs. In contrast, L1 had no effect on endothelial cell morphology or CRP expression. The L5-induced local secretion of CRP may contribute to endothelial cell damage by means of multiple mechanisms. CRP enhances the release of soluble LOX-1 from macrophages by activating TNF-α converting enzyme [40]. Although it has been reported that CRP may target LOX-1 [18], [31], our results showed that blocking LOX-1 with TS92 had no effect on the internalization of recombinant CRP, even though CRP continued to upregulate LOX-1 expression. Therefore, the possibility remains that CRP and L5 do not share the same binding domain on LOX-1. As previously reported, the Fc gamma receptors CD32 and CD64 may be the primary endothelial cell receptors that mediate CRP internalization [41], [42]. The L5-induced local secretion of CRP may contribute to endothelial cell damage by means of other mechanisms, as well. Grad and colleagues [43] reported that endothelial cells expressing CRP increase platelet adhesion to human endothelial cells. Furthermore, CRP has been shown to promote atherosclerosis formation by inducing endothelial cells to produce monocyte chemoattractant protein-1 [44] and to express cell adhesion molecules [45], [46]. The L5-induced increase in expression of the LOX-1 receptor may result in the increased intake of CRP, as well. CRP enhances the expression of LOX-1 on endothelial cells, which may also be a mechanism by which CRP promotes endothelial dysfunction [31]. In summary, L5 can elevate LOX-1 levels, thereby increasing CRP levels and, in turn, elevating LOX-1 expression. The positive feedback loop among L5, CRP, and the LOX-1 receptor may underlie a novel mechanism of endothelial dysfunction and atherosclerosis.

### L5-stimulated Local CRP and ROS Production via LOX-1 in HAECs

We showed that L5 did not possess high thiobarbituric acid reactant values, in contrast to oxidized LDL [47]; however, L5 functions similarly to oxidized LDL in that it can induce oxidative stress, as shown by its ability to increase levels of superoxide dismutase 1 (SOD1). Furthermore, L5 isolated from the LDL of all hypercholesterolemic patients increased ROS production in HAECs within 20 minutes of exposure and directly increased endothelial endogenous CRP levels within 30 minutes for up to 24 hours. Our results support the idea that L5 induces oxidative stress and local endothelial cell inflammation and is therefore pro-atherogenic.

### Reduced L5 Levels in Hypercholesterolemic Patients Treated with Atorvastatin

We observed a significantly higher L5 percentage in total LDL in hypercholesterolemic patients than in healthy control subjects. This finding provides evidence that L5 plays an important role in the process of atherosclerosis and indicates that L5 may be a candidate biomarker for identifying high-risk cardiovascular patients. Moreover, in hypercholesterolemic patients, we showed that atorvastatin therapy effectively reduced not only the total cholesterol and LDL-C levels but also the L5 percentage in total LDL. Thus, it is conceivable that in cultured endothelial cells, statin drugs may avert endothelial damage by preventing L5-mediated LOX-1 elevation, thereby impeding LOX-1-induced CRP expression. Statins may block LOX-1, suggesting that statins have dual functions in reducing cardiovascular risk factors.

Importantly, in the 2 hypercholesterolemic patients who discontinued statin therapy after 3 months, we observed a rebound in L5 percentage and plasma levels 3 months after discontinuation. This suggests that the benefit of statins on L5/CRP inhibition is not permanent and that discontinuation of statin therapy results in the reoccurrence of elevated L5 levels within a 3-month period. Statin drug therapy may lower the risk of coronary artery disease and provide vascular protection not only by reducing the level of LDL-C but also by targeting and reducing the level of circulating atherogenic L5, which in turn lowers circulating CRP levels and local endothelial CRP expression. Consequently, statins may also decrease ROS-initiated oxidative damage, including ROS-generated mitochondrial dysfunction and DNA damage. Given the unique qualities of L5, measurement of the L5 level may emerge as a revolutionary method for assessing the quality of LDL. In patients with dyslipidemia, L5 levels may indicate relative atherosclerotic risk and guide the selection of high-risk patients who are candidates for statin therapy, thereby maximizing the therapeutic benefit of statins in these patients while minimizing statin exposure in low-risk individuals. However, these possibilities need further investigation.

Our findings suggest that in addition to its lipid-lowering effects, atorvastatin also has anti-inflammatory properties. Previous studies have shown that in addition to reducing hepatic LDL secretion through the inhibition of HMG-CoA reductase, statins also inhibit HMG-CoA reductase within smooth muscle cells, monocytes, macrophages, and endothelial cells [48], [49]. Statins were also shown to confer rapid benefits in patients with acute coronary syndrome by blocking pathologic factors such as CRP, tissue factor, IL-1, IL-6, and other pro-inflammatory cytokines [50]. In the PROVE IT–TIMI 22 (Pravastatin or Atorvastatin Evaluation and Infection Therapy–Thrombolysis in Myocardial Infarction 22) trial, atorvastatin (80 mg/day) reduced both LDL and CRP levels at 30 days and at 4 months in patients with acute coronary syndrome, and the early benefit of statins was correlated with the reduction in CRP rather than in LDL levels [27]. In the JUPITER (Justification for the Use of Statins in Primary Prevention: An Intervention Trial Evaluating Rosuvastatin) trial, rosuvastatin was shown to reduce the major cardiovascular events in individuals with relatively low LDL (<130 mg/dL) but high CRP (>2 mg/L) levels. In addition, the mean CRP level in these individuals was 37% lower after 12 months of statin treatment [51]. Because atherosclerosis is regarded as an inflammatory disease, statins were speculated to benefit high-risk cardiovascular patients by reducing systemic levels of CRP. On the basis of results from previous studies, our findings provide a more complete picture of the mechanism for how statin therapy may slow atherosclerosis progression. Because this is a pilot single-center observational study with a small sample size, these results should be confirmed in large-scale studies.

## Conclusion

Atorvastatin lowers the risk factors for coronary artery disease by not only reducing total LDL cholesterol levels but also by reducing circulating levels of electronegative L5 LDL, thereby impeding L5-induced endothelial CRP and ROS production.

## Supporting Information

Figure S1 **Cytotoxicity of L1-L5 in human aortic endothelial cells (HAECs).** The cytotoxicity of LDL subfractions L1-L5 was determined by using Hoechst 33342 nuclear staining. HAECs were treated with 50 µg/mL each of L1-L5 for 24 hours. Only L5 induced significant apoptosis of HAECs, as shown by nuclear condensation and DNA fragmentation. Phosphate-buffered saline was used as a control (Ctrl).
(TIF)

Figure S2 **Effects of L5 on oxidative stress marker superoxide dismutase 1 (SOD1) levels and analysis of L5 oxidation.** (A) Western blot analysis of human aortic endothelial cell (HAEC) total protein lysates shows that L5 increased superoxide dismutase 1 (SOD1) levels after 24 h when compared with phosphate-buffered saline (control, presented as L5 concentration 0). L5 concentrations are expressed in µg/mL. (B) Thiobarbituric acid reactive substances (TBARS) assay results showing that malondialdehyde (MDA) values for L1 and L5 from 4 different patients (#1-#4) were much lower than those of copper-oxidized LDL, used as positive control.
(TIF)

Figure S3 **Analysis of TNFα, p-p38, and NF-κB expression in human aortic endothelial cells (HAECs) treated with L5.** Western blot showing the expression of various inflammatory biomarkers in HAECs treated with 50 µg/mL L5 for 24 hours. L5 notably increased TNFα, p-p38, and NF-κB expression. HAECs were treated with L1 as a control (ctrl).
(TIF)
