# Supplementary figures and images for: Electronegative Low-Density Lipoprotein Increases C-Reactive Protein Expression in Vascular Endothelial Cells through the LOX-1 Receptor
Source: PLoS One. 2013 Aug 8;8(8):e70533. doi: 10.1371/journal.pone.0070533 (PMC3738565; doi:10.1371/journal.pone.0070533)

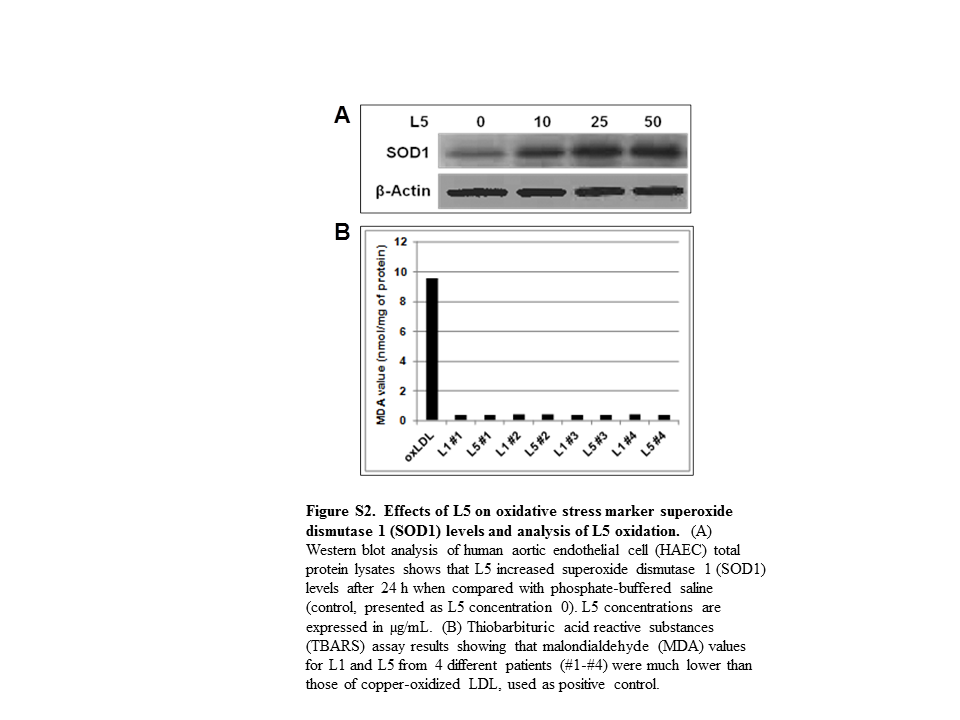

Supplement: Figure S2 — Effects of L5 on oxidative stress marker superoxide dismutase 1 (SOD1) levels and analysis of L5 oxidation. (A) Western blot analysis of human aortic endothelial cell (HAEC) total protein lysates shows that L5 increased superoxide dismutase 1 (SOD1) levels after 24 h when compared with phosphate-buffered saline (control, presented as L5 concentration 0). L5 concentrations are expressed in µg/mL. (B) Thiobarbituric acid reactive substances (TBARS) assay results showing that malondialdehyde (MDA) values for L1 and L5 from 4 different patients (#1-#4) were much lower than those of copper-oxidized LDL, used as positive control. (TIF) [file pone.0070533.s002.tif]

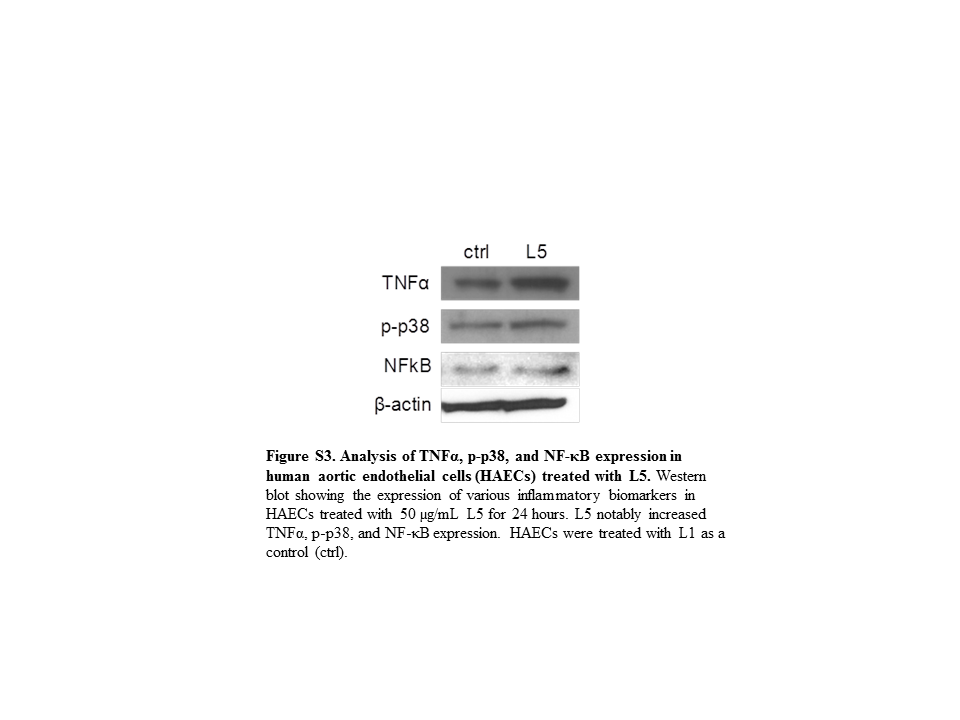

Supplement: Figure S3 — Analysis of TNFα, p-p38, and NF-κB expression in human aortic endothelial cells (HAECs) treated with L5. Western blot showing the expression of various inflammatory biomarkers in HAECs treated with 50 µg/mL L5 for 24 hours. L5 notably increased TNFα, p-p38, and NF-κB expression. HAECs were treated with L1 as a control (ctrl). (TIF) [file pone.0070533.s003.tif]
